# On the Reliability and Repeatability of Surface Electromyography Factorization by Muscle Synergies in Daily Life Activities

**DOI:** 10.1155/2018/5852307

**Published:** 2018-11-22

**Authors:** Juri Taborri, Eduardo Palermo, Zaccaria Del Prete, Stefano Rossi

**Affiliations:** ^1^Department of Economics, Engineering, Society and Business Organization (DEIM), University of Tuscia, Via Del Paradiso, 47, Viterbo 01100, Italy; ^2^Department of Mechanical and Aerospace Engineering, Sapienza University of Rome, Via Eudossiana, 18, Roma 00184, Italy

## Abstract

Muscle synergy theory is a new appealing approach for different research fields. This study is aimed at evaluating the robustness of EMG reconstruction via muscle synergies and the repeatability of muscle synergy parameters as potential neurophysiological indices. Eight healthy subjects performed walking, stepping, running, and ascending and descending stairs' trials for five repetitions in three sessions. Twelve muscles of the dominant leg were analyzed. The “nonnegative matrix factorization” and “variability account for” were used to extract muscle synergies and to assess EMG goodness reconstruction, respectively. Intraclass correlation was used to quantify methodology reliability. Cosine similarity and coefficient of determination assessed the repeatability of the muscle synergy vectors and the temporal activity patterns, respectively. A 4-synergy model was selected for EMG signal factorization. Intraclass correlation was excellent for the overall reconstruction, while it ranged from fair to excellent for single muscles. The EMG reconstruction was found repeatable across sessions and subjects. Considering the selection of neurophysiological indices, the number of synergies was not repeatable neither within nor between subjects. Conversely, the cosine similarity and coefficient of determination values allow considering the muscle synergy vectors and the temporal activity patterns as potential neurophysiological indices due to their similarity both within and between subjects. More specifically, some synergies in the 4-synergy model reveal themselves as more repeatable than others, suggesting focusing on them when seeking at the neurophysiological index identification.

## 1. Introduction

Several locomotive activities, such as walking, running, and ascending and descending stairs, are commonly and continuously performed in daily life [1]. Though perceived as simple, these activities involve the coordination of a high number of muscles of lower limbs [2]. It is generally understood that the central nervous system (CNS) can reduce the dimensionality of neural activation outputs to control muscles and achieve a predefined movement [3, 4]. The reduction is obtained through the simultaneous coactivation of muscle groups, addressed as muscle synergies [5, 6]. Parameter estimation of muscle synergy models is usually obtained from the factorization of the electromyographic (EMG) signals by using decomposition algorithms [7]. The nonnegative matrix factorization [8] is the most widespread, even though other methods, such as principal or independent component analysis and inverse Gaussian, can lead to similar results [7, 9].

The factorization of the EMG signals by means of the muscle synergy model has been used in several tasks, such as locomotion [9, 10], balance [11, 12], reaching [13, 14], or sport gestures, such as cycling, bench press, and rowing [15–17]. Nowadays, the goal of using EMG or muscle synergy parameters as neurophysiological indices, in clinical routine [18–20], sport [17, 21], and robot control [22–25], is gaining more and more appeal [26]. As regards clinical routine, Rodriguez et al. [19] demonstrated that patients with Parkinson's disease adopted a simplified muscle control, i.e., a lower number of muscle synergies, during walking with respect to healthy subjects, while Shuman et al. [18] provided a comparison between children with cerebral palsy (CP) and age-matched controls, assessing a lower number of muscle synergies in the experimental group. A lower number of muscle synergies was also observed in poststroke patients [27, 28].

In sport field, Frère and Hug [21] showed that 3 synergies are sufficient to explain the EMG signals during backward giant swing on the high bar performed by expert gymnasts. Kristiansen et al. [17] found that the CNS has the capability to activate only two muscle synergies during bench press exercises in bodybuilders. As an example of cross-contamination in the robotic field, Artemiadis and Kyriakopoulos [22] and Lunardini et al. [23] used muscle synergies to control a robot for the rehabilitation of the upper limbs, in adults and children with CP, respectively.

To verify that muscle synergies are not a mere output of a mathematical approach but a useful tool to understand the organization of CNS in the achievement of a motor task, several studies investigated the methodological issues related to EMG factorization. In particular, Steele et al. [29] evaluated the effects of both the number and the specific examined muscles, finding that the two variables affect the structure of muscle synergies. Tresch and colleagues [7], instead, demonstrated that different factorization algorithms allow reaching equal results, as also confirmed by Santuz et al. [9]. The influence of filter parameters required for the EMG preprocessing on the muscle synergies was demonstrated considering both the repeatability [9] and the accuracy [30] of the selected number of muscle synergies. Finally, Oliveira et al. [31] focused their study on the effects of averaging or concatenating repetitions of the same task, assessing that the average allows obtaining a higher reconstruction quality even though it neglects the contribution of the step-to-step variability. In addition, to evaluate the viability of using muscle synergy parameters as neurophysiological indices, the robustness in the muscle synergy extraction needs to be deeply investigated. An excellent within-subject repeatability, both within and between days, has been already demonstrated during walking trials of children with cerebral palsy [18] and during walking and running trials of healthy subjects [9]. As regards the between-subject repeatability, it has been showed to be good in bench press exercises [17] and pedaling trials [15]. All the abovementioned papers have some methodological limitations since they did not quantify (i) the within- and between-subject repeatability in tasks different to the gait involving daily life activities, such as stepping in place and ascending and descending stairs, that could be implemented as useful scenarios in the control of lower limb robotic devices and (ii) the within- and between-subject robustness of the EMG reconstruction by means of a specific muscle synergy model considering both the overall reconstruction and each muscle individually. To the best of authors' knowledge, all these methodological implications have not been evaluated. A thorough investigation is mandatory before validating the neurophysiological parameters based on the muscle synergy extraction and to give insightful information to researchers involved in the control design of lower limb robotic devices.

The aim of the present study is to investigate the reliability of muscle synergy theory, following two crucial questions still untapped. Firstly, we investigated the robustness of the EMG reconstruction in within- and between-subject analysis, focusing on both the overall reconstruction and the reconstruction of each single muscle. Robustness of EMG reconstruction, in fact, is a mandatory requirement when using EMG factorization in clinical, robotic, and sport applications [9, 26]. Secondly, we assessed the within-subject repeatability, i.e., both within and between sessions, and the between-subject repeatability of the parameters provided by the muscle synergy model in several daily life activities, to evaluate the viability of muscle synergy parameters as robust neurophysiological indices.

## 2. Materials and Methods

### 2.1. Experimental Protocol

Eight healthy volunteers (three males and five females, age: 27.8 ± 3.8 years, height: 1.72 ± 0.12 m, mass: 61.5 ± 14.9 kg) were enrolled in the experimental protocol. Subjects have never had known neuromuscular and vestibular pathologies. A written informed consent was obtained from the participants according to the ethical standards outlined in the 1964 Declaration of Helsinki.

The protocol consisted of five tasks: walking (W), stepping in place (S), running (R), ascending stairs (A), and descending stairs (D). W and R were performed through a pathway of 15 m at the self-selected speed, and the acquisition was stopped at the end of the pathway after one passage. S task lasted for 15 s, and each subject was free to choose the preferred cadence. In A and D tasks, subjects were asked to perform only one ascent (A) or descent (D) per repetition using a staircase with 20 steps. All tasks were repeated five times in barefoot condition. The entire protocol was repeated for three different sessions, separated each other by at least 24 h. Consequently, each subject performed a total of 75 trials that are five repetitions of five tasks for three sessions.

**Figure 1:**
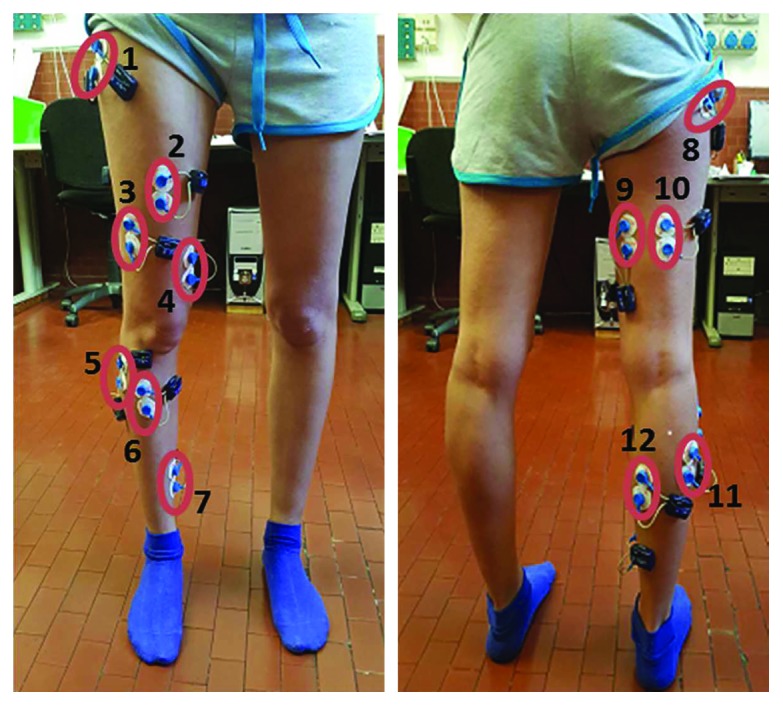
Muscles recorded during the experimental protocol from the dominant leg of each subject: example of electrode placement. 1: tensor fasciae latae (TFL); 2: rectus femoris (REF); 3: vastus lateralis (VLAT); 4: vastus medialis (VMED); 5: peroneus longus (PERO); 6: tibialis anterior (TA); 7: soleus (SOL); 8: gluteus maximus (GLU); 9: semitendinosus (SEMT); 10: biceps femoris (BIF); 11: gastrocnemius lateralis (LGAS); 12: gastrocnemius medialis (MGAS).

The activity of 12 muscles, shown in Figure 1, was recorded from the dominant leg, identified as the one used to kick a ball [32]. All the subjects were right dominant. The placement of passive surface Ag/AgCl circular electrodes (BlueSensor M, Ambu, Ballerup, Denmark) in differential configuration was performed by a skilled operator, the same for all subjects, according to the SENIAM guidelines [33]. Following these guidelines, the maximum electrode size in the direction of the muscle fibers is equal to 9 mm. In addition, these guidelines ensure placing electrodes far enough from the innervation zone, which can influence the recorded signals. Such recommendations were chosen as SENIAM is a recognized association in EMG research [34]. The repetitions within the same session were performed without removing the electrodes, while electrode replacement was performed between sessions. No measures were conducted to ensure the same positioning neither a marker on the subject was drawn among different sessions. Thus, the potential effects of intraoperator electrode replacement have been taken into account as a variable for muscle synergy parameter repeatability.

The foot of the dominant side was equipped with two footswitches (Footswitch FSR sensors, Wave, Cometa, Italy) underneath the toe and the heel. EMG and footswitch signals were synchronously acquired at 2000 Hz via a wireless system for electromyography (Wave, Cometa, Milan, Italy).

### 2.2. Data Processing

The footswitch status (pressed/not pressed) was used to identify the strides in W and R tasks and the single event in S, A, and D tasks, both defined as the interval between two consecutive heel strikes of the same foot. In order to avoid the influence of acceleration and deceleration phases and consider the same number of cycles for all tasks, only data corresponding to eight strides in W and R or steps in S, A, and D were selected from the acquired signals. In particular, for W and R tasks, the eight strides are the ones in the center of the acquired signal. In addition, the footswitch outputs were used to compute the cadence, expressed in steps/min, related to each subject in each repetition and session, independently for each task. Then, mean and SD were computed for each task across subjects and sessions. The ratio between SD and mean, expressed as percentage, allows calculating the coefficient of variation (CoV), quantifying the variability of the cadence in each task.

Following the methodological guidelines proposed by Santuz et al. [9], the mean value was removed from EMG signals and a high-pass filter at 50 Hz was applied. EMG signals were rectified and low-pass filtered at 20 Hz, to extract the envelope. All filters were 4th-order, zero-phase, Butterworth type. Negative values in the resulting signal due to a low-pass filter overshoot were artificially set to zero. The envelope signal was then divided into the eight previously identified strides based on footswitch data, interpolated to 1000 frames, and averaged across the strides, to obtain a higher reconstruction quality [31]. The amplitude of the EMG vector was normalized with respect to the maximum activation, defined as the maximum value found in a set including all tasks and all sessions. Thus, a single value for each muscle was used for the normalization process as suggested by de Marchis et al. [35]. An EMG matrix (*m* × *n*) was obtained for each task, each repetition, and each session by grouping the *n*-sample signals of the *m* considered muscles by row. In our case, *m* was equal to 12 and *n* was equal to 1000.

The extraction of muscle synergy parameters from EMG matrix was performed via the nonnegative matrix factorization (NNMF), which allows decomposing the EMG signals into muscle synergy vectors (**W**_*i*_) and temporal activity patterns (**C**_*i*_), according to a linear combination, as in the following equation [8]:

(1) $$\begin{array}{c} EMG=\overset{s}{\underset{i=1}{\sum }}W_{i}C_{i}+residual,\;s\leq m, \end{array}$$

where *s* represents the number of muscle synergies in the tested model and the residual was considered as the difference between the acquired EMG matrix and the reconstructed one. Briefly, each **W**_*i*_ is a time-invariant vector, composed by positive weights, indicating the relative contribution of each muscle to the *i*-th synergy. Each **C**_*i*_ is a time-variant waveform vector hypothesized as the neural command for the activation of the *i*-th synergy.

Equation (1) can provide multiple solutions for **W**_*i*_ and **C**_*i*_, once the number *s* of synergies in the model is selected. We solved the equation 12 times, increasing *s* from 1 to *m*, using the following parameters: 50 replicates and 1000 maximum iterations for minimizing squared residual between acquired and reconstructed signals [18]. We performed the NNMF in MATLAB (2012b, MathWorks, Inc., Natick, Massachusetts, United States).

### 2.3. Data Analysis

The variability account for (VAF) was chosen to assess the similarity between the acquired and reconstructed EMG signals [36], computed for all tested numbers of synergies. VAF was computed as uncentered Pearson's coefficient, expressed as percentage. In particular, Pearson's correlation coefficient between the acquired and the reconstructed EMG matrices was used to compute the global VAF (VAF_glo_), while the local VAF (VAF_loc_) was calculated by correlating the acquired and the reconstructed EMG signal of each muscle.

For each subject, task, repetition, and session, we selected the model with the minimum number of synergies (NoS), which simultaneously met two selection criteria: VAF_glo_ ≥ 90% and VAF_loc_ ≥ 75% [15, 36]. Range of NoS for each subject across the three sessions was calculated, and median value and statistic frequency of each NoS were computed across all the repetitions and subjects, individually for each task. Thus, the potentiality of the NoS to be considered as a neurophysiological index is assessed focusing on its repeatability both within and between subjects in all examined tasks.

Focusing only on the most common model selected by the subjects considering all the examined tasks, we evaluated the repeatability of the muscle synergies considering three analyses: (i) *within-subject-within-session* (WW), to quantify the robustness of the muscle synergy extraction with respect to the variability induced by different muscle activations of the same subject to achieve the same motor task; (ii) *within-subject-between-sessions* (WB), to quantify the robustness with respect to the variability of the muscle activation of the same subject due to electrode replacement; and (iii) *between-subjects* (B), for quantifying the robustness of the algorithm with respect to different muscle activations by different subjects to achieve the same motor task.

As regards the WW analysis, mean and SD of the VAF_glo_ and VAF_loc_ were computed individually for each subject and each session. Then, the minimum and maximum of the mean value and their standard deviations (SDs) across subjects and sessions were selected for each task. SDs quantify the robustness of reconstruction goodness of EMG data gathered from the same subject in the same session. Moreover, to assess the robustness of the **W**, we computed the cosine similarity (cos_sim_), which is the ratio between the scalar product and the product of the Euclidian norms of two vectors, as in the following equation [9]:

(2) $$\begin{array}{c} \cos_{sim}\left(W_{x},W_{y}\right)=\frac{W_{x}\cdot W_{y}}{\left\|W_{x}\right\|\left\|W_{y}\right\|}, \end{array}$$

where **W**_*x*_ and **W**_*y*_ represent the pair of tested **W** in turn. This index ranges from 0 to 1, corresponding to no and perfect similarity, respectively. The threshold value for cos_sim_ to assume similarity was set to 0.60 [3]. While to assess the similarity of the **C**, we computed the coefficient of determination (*R*^2^) as follows [9]:

(3) $$\begin{array}{c} R^{2}=1-\frac{\sum_{i=1}^{n}{\left(C_{x_{i}}-C_{y_{i}}\right)}^{2}}{\sum_{i=1}^{n}{\left(C_{x_{i}}-\bar{C_{x}}\right)}^{2}}, \end{array}$$

where **C**_*x*_ and **C**_*y*_ represent the pair of tested **C** in turn. *R*^2^ ranged from −∞ to 1, where 1 indicates the perfect similarity and 0.70 is the threshold to assess the similarity [9].

Considering that the order of synergies in the model obtained via NNMF is not consistent among different runs of the algorithm [18], we performed a *K*-means cluster analysis to select and to order similar synergies between repetitions, sessions, and subjects for each task [37]. In particular, the most correlated synergy vectors that fell within one cluster according to the *K*-mean outputs were considered related to the same *i*-th synergy in the selected model between repetitions, sessions, and subjects for each task.

For WW, ^WW^cos_sim_ and ^WW^*R*^2^ values were obtained, respectively, by pairwise comparisons of the **W** and of the **C** related to the five repetitions in the same session. Thus, we performed 10 comparisons for each subject, each session, each task, and each synergy in the selected model. Mean and SD of ^WW^cos_sim_ and of ^WW^*R*^2^ across the comparisons were computed; then, minimum and maximum and related SDs across subjects and sessions were selected. Each SD was used to quantify the robustness of both the muscle synergy vectors **W** and the temporal activity patterns **C** in the same session performed by the same subject. Moreover, mean and SD of the overall model were computed by independently averaging the ^WW^cos_sim_ and ^WW^*R*^2^ related to all muscle synergies.

As concerns WB analysis, mean and SD of the VAF_glo_ and VAF_loc_ were computed individually for each subject, considering the values obtained during all the sessions together. Then, the minimum and maximum of the mean value and the related SDs across subjects were evaluated for each task. SDs quantify the robustness of the reconstruction goodness of the EMG data gathered from the same subject in different sessions. As regards the repeatability of the muscle synergy vectors and the temporal activity parameters, we firstly averaged the **W** and the **C** related to the five repetitions of each session, obtaining three **W** and the **C**. Then, ^WB^cos_sim_ and ^WB^*R*^2^ values were computed by pairwise comparisons of the three **W** and of the three **C**. Thus, we performed three comparisons for each subject, each task, and each synergy in the selected model. Then, mean and SD of ^WB^cos_sim_ and of ^WB^*R*^2^ across the comparisons were computed, and minimum and maximum and related SDs across subjects were evaluated. These parameters quantify the robustness of the muscle synergy vectors **W** and temporal activity patterns **C** in different sessions performed by the same subject. Moreover, mean and SD of the overall model were computed by independently averaging the ^WB^cos_sim_ and the ^WB^*R*^2^ related to all muscle synergies.

Finally, for the B analysis, mean and SD of the VAF_glo_ and VAF_loc_ were computed considering the values obtained during all the sessions and all subjects together. SDs were used to quantify the robustness of the reconstruction goodness of the EMG data gathered from different subjects in different sessions. In addition, to compute ^B^cos_sim_ and ^B^*R*^2^ values, we firstly averaged the **W** and the **C** of the three sessions individually for each subject and then, we performed pairwise comparisons among subjects using the obtained mean **W** and mean **C**. Thus, we performed 28 comparisons for each task and each synergy in the selected model. Then, mean and SD of ^B^cos_sim_ and of ^B^*R*^2^ across the comparisons were computed to quantify the robustness of the muscle synergy vectors **W** and the temporal activity pattern **C** in different sessions performed by different subjects. Moreover, mean and SD of the overall model were computed by independently averaging the ^B^cos_sim_ and the ^B^*R*^2^ related to all muscle synergies.

Considering the results of the ^WW^cos_sim_, ^WB^cos_sim_, ^B^cos_sim_, ^WW^*R*^2^, ^WB^*R*^2^, and ^B^*R*^2^, we assessed the potentiality of both the **W** and **C**, related to the overall composition and to the *i*-th synergy in the selected muscle synergy model, to be considered as neurophysiological indices.

### 2.4. Statistical Analysis

To assess the reliability of the method, ICC was computed both on the VAF_glo_ and VAF_loc_ for each task, considering all sessions together. ICC values in the range 0.00–0.39 were classified as poor, 0.40–0.59 as fair, 0.60–0.74 as good, and 0.75–1.00 as excellent [38].

All data, with the exception of the NoS, were tested for normality with the Shapiro-Wilk test, and they resulted normally distributed. For all the following test, a significance level equal to 0.05 was set.

A Kruskal-Wallis nonparametric test was performed to evaluate statistical differences in the NoS, considering the task as the independent variable. If statistical differences were found, Dunn's test was conducted to analyze where the differences were present.

One-way repeated measurement ANOVA tests were conducted for mean values of the ^B^VAF_loc_, considering muscles as the independent variable. The Greenhouse-Geisser correction was adopted if the assumption of sphericity was violated. Bonferroni's test for multiple comparisons was performed when statistical differences were found.

The influence of the *i*-th synergy on ^B^cos_sim_ and ^B^*R*^2^ in the selected synergy model was tested through one-way ANOVAs, independently for each task. When the statistical differences were found, Bonferroni's test for multiple comparisons was performed.

Statistical analysis was conducted using SPSS software package (IBM-SPSS Inc., Armonk, NY, USA).

## 3. Results

The following cadence values were obtained by processing the footswitch outputs: (i) 120 ± 10 steps/min with a variability equal to 8.3% for W, (ii) 110 ± 8 steps/min with a variability equal to 7.2% for S, (iii) 155 ± 15 steps/min with a variability equal to 9.7% for R, (iv) 100 ± 6 steps/min with a variability equal to 6.0% for A, and (v) 102 ± 9 steps/min with a variability equal to 8.8% for D.

As regards NoS, the ranges for each subject related to all tasks were reported in Table 1. Taking into account the three sessions, no subject recruited the same muscle synergy model during all examined repetitions related to each task. Considering all subjects, NoS ranged from 3 to 6 and the median value is always equal to 4 for all the tested daily life activities. However, some statistical differences were found among the activities: W vs. S, W vs. R, S vs. R, S vs. A, R vs. A, and R vs. D (always *p* < 0.01).

**Figure 2:**
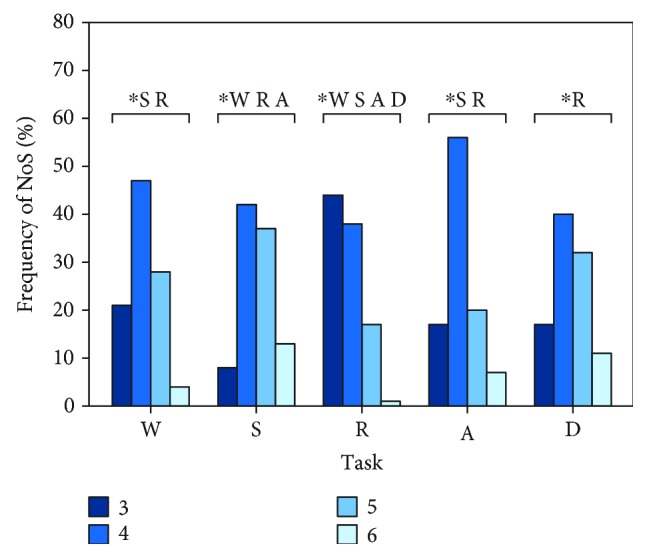
Frequency of occurrence of the synergy model for each daily life activities. ∗ stands for statistical differences.

**Figure 3:**
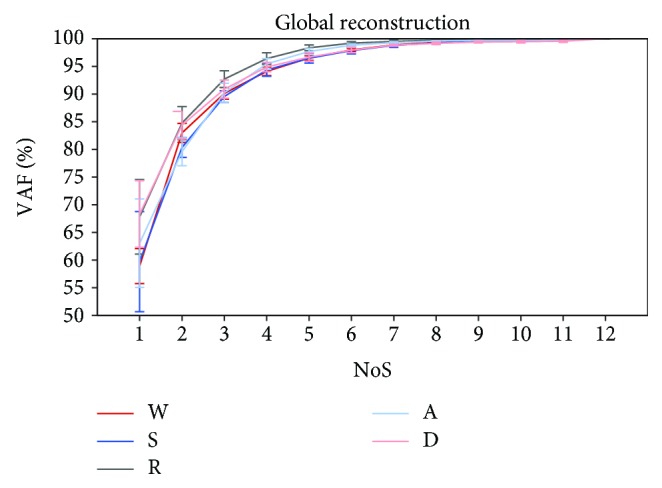
Mean and standard deviation of the VAF_glo_ across the subjects as function of the number of muscle synergies for each task.

In Figure 2, the frequencies of the selected synergy model for all tasks are shown. The most frequent model was the 4-synergy one for all tasks, with the exception of running (3 synergies). Moreover, it was also found that the 6-synergy model was the less likely selected for all tasks since it was the one associated with the minimum value of frequency occurrence, with the exception of stepping in place (3 synergies). Means and SDs of the VAF_glo_ as function of the number of synergies for each task are reported in Figure 3. To compare the results across the daily life tasks, we focused only on the 4-synergy model, as it resulted the most selected model considering all the subjects and all the tasks together.

Focusing on reliability analysis, ICC values of the VAF_glo_ and VAF_loc_ among sessions are reported in Table 2. The methodology showed an excellent reliability for the global reconstruction in all tasks. Considering each muscle, reliability ranged from fair to excellent in walking and from good to excellent in the other tasks.

Taking into account the within-subject-within-session (WW) analysis, ^WW^VAF_glo_ and ^WW^VAF_loc_ values were always above 90.4% and 71.6%, respectively (Table 3). The highest values of SD were 1.2% and 9.7% for the global and the local reconstructions, respectively. As reported in Table 4, ^WW^cos_sim_ ranged from 0.71 to 0.99 considering all synergies and all activities, while a SD up to 0.24 was found. The highest overall mean value, averaging all synergies in the model, was related to A, i.e., 0.94, while the lowest one was related to D, i.e., 0.89. Taking into account the similarity related to the temporal activity patterns, ^WW^*R*^2^ ranged from 0.80 to 0.99 considering all synergies and all tasks, while the maximum value of SD was 0.10 (Table 5). The highest overall mean value was related to the R (0.94) and the lowest one to the D (0.91).

**Figure 4:**
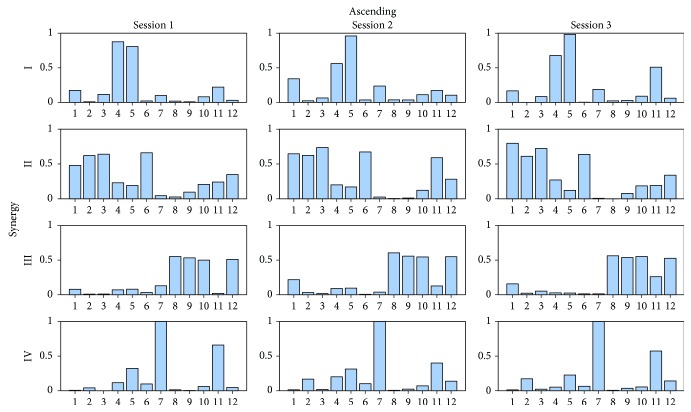
A paradigmatic example of the mean value of muscle synergy vectors in the 4-synergy model related to the ascending task for one subject for each session. The mean has been obtained averaging the repetitions of the same session. For visualization purposes, muscle synergy vectors are normalized to unity. Muscle numbers: 1: TFL; 2: REF; 3: VLAT; 4: VMED; 5: PERO; 6: TA; 7: SOL; 8: GLU; 9: SEMT; 10: BIF; 11: LGAS; 12: MGAS.

**Figure 5:**
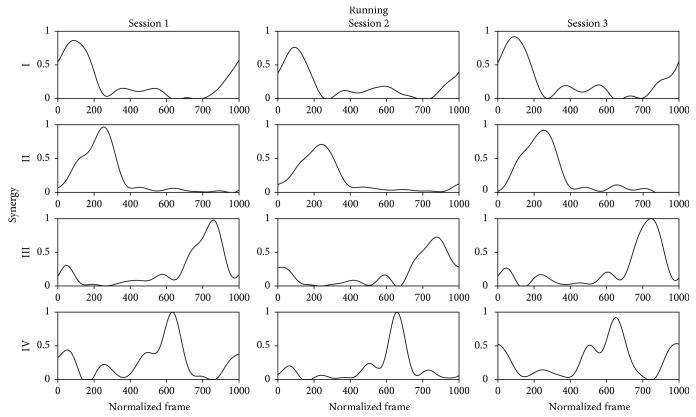
A paradigmatic example of the mean value of the temporal activity pattern in the 4-synergy model related to the running task for one subject for each session. The mean has been obtained averaging the repetitions of the same session. For visualization purposes, temporal activity patterns are normalized to unity.

As concern the within-subject-between-session (WB) analysis, a paradigmatic example of the differences of **W** and **C** during the three sessions related to one subject in one task is shown in Figures 4 and 5.  Table 6 reports that the lowest ^WB^VAF_glo_ and ^WB^VAF_loc_ values were 91.9% and 74.2%, respectively, while the highest SD values were 1.9% and 9.8%. By analyzing Table 7, ^WB^cos_sim_ ranged from 0.84 to 0.99 considering all synergies and all activities and a SD up to 0.08 was found. The results obtained by averaging the outcomes of all muscle synergies in the model showed the highest overall mean value of the ^WB^cos_sim_ for the R and A (0.95) and the lowest one for the W (0.92). Focusing on Table 8, ^WB^*R*^2^ ranged from 0.85 to 0.99 with a maximum SD value equal to 0.08. Considering the overall composition, R was associated to the highest value (0.91) and the other tasks to the lowest one (0.90).

**Figure 6:**
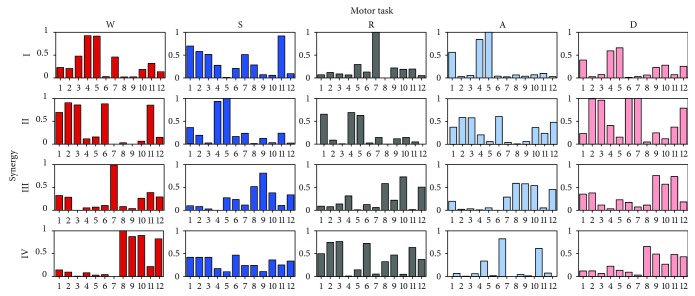
Mean value of muscle synergy vectors in the 4-synergy model related to each daily life activities. The mean has been obtained by averaging across subjects the muscle synergy vector mean of the three sessions of each subject. For visualization purposes, muscle synergy vectors are normalized to unity. Muscle numbers: 1: TFL; 2: REF; 3: VLAT; 4: VMED; 5: PERO; 6: TA; 7: SOL; 8: GLU; 9: SEMT; 10: BIF; 11: LGAS; 12: MGAS.

**Figure 7:**
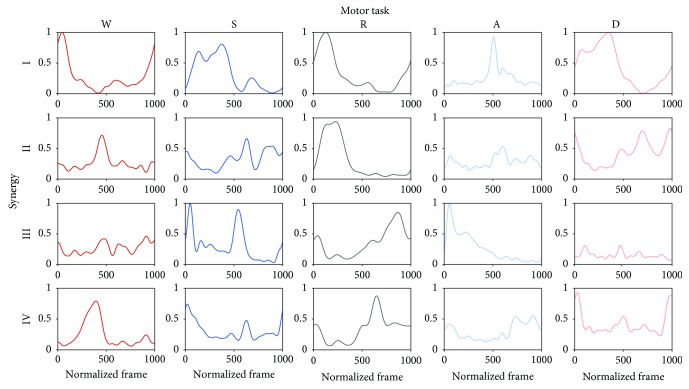
Mean value of the temporal activity pattern in the 4-synergy model related to each daily life activities. The mean has been obtained by averaging across subjects the temporal activity pattern mean of the three sessions of each subject. For visualization purposes, temporal activity patterns are normalized to unity.

Finally, considering the between-subject analysis (B), the mean values of both the muscle synergy vectors **W** and the temporal activity patterns **C** considering all subjects and all sessions are shown in Figures 6 and 7 for each task. The histograms and waveforms are useful to clarify the inner composition of the synergies in the model, necessary to understand the following results in terms of repeatability related to each specific synergy.  Table 9 reports the minimum value of 93.9% and 84.3% for the ^B^VAF_glo_ and ^B^VAF_loc_ and a maximum SD value of 1.7% and 7.6%, respectively. As regards the statistical outcomes, the mean value of ^B^VAF_loc_ related to the GLU was statistically different from the same parameter computed for LGAS (*p* = 0.02) during W task. Statistical differences were found also in S task, between ^B^VAF_loc_ related to TA and MGAS (*p* = 0.05). As regards A task, statistical differences were found between the ^B^VAF_loc_ related to the TFL and the one computed for REF (*p* = 0.02), VLAT (*p* = 0.01), VMED (*p* = 0.01), PERO (*p* = 0.01), TA (*p* = 0.01), SOL (*p* = 0.02), and MGAS (*p* = 0.01) and other differences were observed between ^B^VAF_loc_ related to the GLU and the ones computed for REF (*p* = 0.03), VLAT (*p* = 0.01), VMED (*p* = 0.01), PERO (*p* = 0.04), SOL (*p* = 0.04), and MGAS (*p* = 0.02). No statistical differences were found in R (*p* = 0.17) and D (*p* = 0.18). The ^B^cos_sim_ values were in the range of 0.79–0.90, with a SD up to 0.10 (Table 10). Differences among the specific synergies in the model were found in W (always *p* < 0.01), R (always *p* = 0.01), and D (always *p* < 0.01), as reported in Table 10. By averaging the ^B^cos_sim_ of all muscle synergies in the model, the highest overall mean value was associated to the A (0.86) and the lowest to the S (0.82). Finally, the ^B^*R*^2^ felt in the range 0.84–0.92, with a SD up to 0.08. Differences among the specific synergies in the model were found in all tasks (always *p* < 0.01), as reported in Table 11. By taking into account the overall composition, all tasks reached an ^B^*R*^2^ equal to 0.87, with the exception of D (0.84).

## 4. Discussion

The present study represents an experimental insight on the reliability of synergy-based EMG signal factorization. Specifically, we evaluated the within- and between-subject variability of the reconstruction of the EMG signals associated with the technique. Further, we aimed to assess the repeatability of the muscle synergy parameters, which are number of muscle synergies, goodness of reconstruction, muscle synergy vectors, and temporal activity patterns, both within and between sessions during a set of daily life activities.

### 4.1. Is the EMG Reconstruction Reliable and Repeatable in Within-Subject and Between-Subject Analysis?

Results related to the reliability of the muscle synergy extraction permit to assume that factorization of EMG data via muscle synergies expresses an excellent reliability, considering the global reconstruction of muscle activities during the examined tasks. On the contrary, fair reliabilities related to local reconstruction of some muscles confirm the findings reported by Kristiansen et al. [17], recommending a particular attention to the variability of the method, when handling data from some specific muscles such as biceps brachii, triceps brachii, and the rectus femoris, which is also found to be the one with the lowest value of ICC in this study. These results could be ascribed to the high between-subject variability of the VAF, as already shown by Kristiansen et al. [17].

The walking task showed a lower reliability than the other motor tasks, suggesting that the CNS is able to control these movements by means of different activation patterns, as also reported by De Marchis et al. [39]. Comparing walking with running, the outcomes of lower reliability can be justified considering that the gait cycle becomes less variable as speed increases [40]; instead, comparing walking with the other examined tasks, we speculate that external constraints, such as stairs, cause a decrease of gait variability as also reported by Donath et al. [41].

As regards the repeatability of EMG reconstruction goodness, the high value of the VAF_glo_ and the low SD values proved that the selection criterion on the global reconstruction was always met in both within- and between-subject analyses for all tasks. Thus, the variability induced by the different muscle activations of the same subject, that is, the recruitment of different NoS to perform the same task, does not influence the goodness of the linear combination used for the global reconstruction. Considering these outcomes, we can affirm the robustness of the EMG factorization by means of the 4-synergy model if researches have to pay attention only to the global reconstruction of the EMG activity. By looking to the high SD values related to the VAF_loc_, we can affirm that the selection of the most common synergy model can lead to inaccurate EMG reconstructions related to some specific muscles; in particular, the reconstruction of the EMG signals related to the TFL, TA, and GLU generally appeared to be the one less accurate in all tasks when a 4-synergy model is selected. Finally, the statistical differences found in the mean values of ^B^VAF_loc_ in walking, stepping, and ascending stairs suggest to carefully selecting the muscles to include in the experimental setup, to respect the goodness of the reconstruction required by the specific application. The greater number of statistical differences was found for the gluteus, both in walking and in ascending stairs, and the tensor fasciae latae in the ascending task. These findings can be ascribed to the more relevant soft tissue and cross talk artifacts due to the position of the electrodes [42].

By summarizing the findings related to the reliability and repeatability analysis of the VAF, the extraction of muscle synergy by means of the nonnegative matrix factorization can be used in several applications, requiring robust goodness of EMG reconstruction, such as clinical analysis, sports performance evaluation, or robotics. However, the use of EMG factorization via the muscle synergies is recommended only in applications that require a reliable overall reconstruction of the acquired EMG signals. In fact, when the reconstruction of the single muscle has been required, such as for controlling a robotic device via muscle synergies [22] or monitoring the severity of a pathology [43], the actual reliability and variability of the specific muscle reconstruction have to be considered.

### 4.2. Might Muscle Synergy Parameters Be Considered as Neurophysiological Indices?

Both the range (3–6) and the median value (4) of NoS found in this study for all tasks provide a further confirmation of the hypothesis that the CNS reduces the complexity of muscle activation for achieving a motor task, also in activity never explored before, such as ascending and descending stairs. Concerning the frequency values of NoS, we observed that our results, related to the walking and stepping, are in accordance with the literature [5, 9, 36, 44]. Conversely, a lower dimensionality of the synergy model is observed in the running task with respect to Santuz et al. [9]. In particular, they analyzed the activation of lower and upper body muscles and found a greater number of muscle synergies in running than in walking. This difference could be ascribed to the reduced number of muscles that we analyzed, as we neglected those in the upper body. It is worth noticing that synced arm movements are necessary to keep balance in running [45]. Thus, we speculate that the absence of upper body muscles in the experimental setup could explain how the dimensionality of our model is in contrast with literature for running task, but in accordance for walking and stepping, where the recruitment of upper body muscles is less crucial for the body balance. Moreover, statistical differences on the median value found for the NoS in running compared with those in the other tested daily life activities suggest that the EMG signals gathered during running can be reconstructed with a lower number of synergies. The absence of a frequency of NoS equal to 100% in all tasks, both within and between sessions for healthy subjects with similar demographic characteristics, might be ascribed to the capability of the CNS to generate different activation profiles for reaching the same motor task, resulting in a different number of muscle synergies [39]. The ability of NoS to discriminate between healthy subjects and patients with neurological diseases was previously assessed in patients with cerebral palsy [18] and Parkinson's disease [19]. In particular, the authors found a maximum spread of 2 NoS between the control and patient groups in walking tasks. Our findings showed a 2 NoS difference also in within-subject and between-subject analyses performed on a healthy population. Consequently, the NoS lacks in the sufficient repeatability level to be considered a robust neurophysiological index.

Focusing on the weights of **W**, instead, we observed that ^WW^cos_sim_ was always above the threshold value of 0.60 for each synergy and for the overall model; however, the variability related to some synergies is not negligible. In fact, SDs indicated that cos_sim_ values spanned also under the similarity threshold of 0.60, considering a confidence interval of 95.5%, i.e., mean value ± 2SD. The variability in the muscle synergy vectors could be addressed to the CNS capability to generate different within-subject activation profiles to achieve the same motor task [15]. It confirms our findings related to the different NoS to perform the same task by the same subject. Moving to the ^WB^cos_sim_, the variability can be considered negligible since the SD values related to each synergy and the overall model were above the similarity threshold also considering the confidence interval of 95.5%. By summarizing, the variability of the cos_sim_ in within-session analysis implies also values lower than 0.60; thus, our results suggest caution in assuming that the weights related to the *i*-th muscle synergy are consistent across repetitions of the same task performed by the same subject if an average across the repetitions is not conducted. Similar repeatability with respect to the one observed by Santuz et al. [9] for the intra- and interday repeatability in walking and running was observed in this study. Thus, we can speculate that the use of a treadmill, as in [9], does not influence the walking and running patterns [46]. As regards the ^B^cos_sim_, the mean values were lower than those evaluated in the WW and WB analyses. These findings can be mainly ascribed to the well-known between-subject variability of the EMG signals [47]. As an alternative hypothesis, they could be due to the variability of the biomechanical data in the execution of the same task by different subjects. However, such effect appeared limited, as shown by the variability of the cadence always lower than 10%. However, some *i*-th synergies and the overall composition of the muscle synergy vector reached mean values above the similarity threshold also with a confidence of 95.5%, affirming themselves repeatable across subjects and potentially useful as neurophysiological indices. The only exception of this finding was the IV synergy related to the stepping task. From the statistical results, it emerged that the third for walking and descending stairs and the fourth for running can be considered the most repeatable synergy vectors related to each motor task. On the contrary, the absence of significant differences in stepping and in ascending stairs did not allow selecting only one **W** as the most repeatable. We can conclude that the muscle synergy vectors related to some specific synergy in the 4-synergy model can be considered acceptable as neurophysiological indices due to demonstrated similarity across a cohort of subjects having similar demographic characteristics and health condition. In addition, it is worth noticing that in W, D, and R, it is suggested to use only the most repeatable **W**.

The same trend of the muscle synergy vectors can be observed for the repeatability analysis related to the temporal activity parameters by considering the WW and WB. In fact, ^WW^*R*^2^ and ^WB^*R*^2^ values are always above the threshold value of 0.70 for each synergy and the overall model, but the value of some SD related to ^WW^*R*^2^ indicated that some comparisons in WW analysis did not reach the set threshold if a confidence level of 95.5% is considered. The found values are in line with the one obtained by Santuz et al. [9]. As for the **W**, the variability in the temporal activity patterns suggests that the CNS can organizes different muscle activations to achieve the same motor task [15]. Thus, our results suggest caution in assuming that the each **C**_*i*_ in the 4-synergy model is consistent across repetitions of the same task performed by the same subject in the same session. By moving to the ^B^*R*^2^, all the values related to each synergy and to the overall model were above the similarity threshold also considering a confidence interval at 95.5%, with the exception of the third synergy for walking and the first synergy for the stepping. However, by considering the statistical outcomes, we can select the neurophysiological index in the most repeatable temporal activity pattern for each task, in particular, the one related to the second synergy for stepping and running and the one related to the first synergy for the descending stairs. A further selection to identify the most repeatable **C** between the one related to the first and second synergies in W and among the one related to the first, third, and fourth synergies in ascending stairs cannot be performed with the found statistical outcomes. In conclusion, we can state that also the temporal activity patterns related to some specific synergy in the 4-synergy model can be assumed as neurophysiological indices due to the promising results related to their repeatability. As for **W**, the selection of the most repeatable **C** is preferred in S, R, and D.

Thus, we can conclude that both the **W** and the **C** can be considered as feasible neurophysiological indices due to the verified good repeatability in within- and between-subject analysis. It is worth noticing that the verified repeatability has two different meanings: the repeatability of the **W** indicates that the weighting of the examined muscles in the *i*-th synergy is similar across subjects, while the repeatability of the **C** shows that the neuromotor signal modulating the excitation of the *i*-th synergy is consistent across subjects [16].

## 5. Conclusion

In this paper, we analyzed the reliability and repeatability of the EMG factorization by means of muscle synergy theory in several daily life activities, such as walking, running, stepping, and ascending and descending stairs. These analyses have been conducted both within and between subjects. Actually, the final goal of the study is to identify the most potential neurophysiological index among the muscle synergy parameters. The outcomes of our study endorse muscle synergy factorization as a robust tool to interpret the CNS activation model, as the repeatability can be mainly affected by the intrinsic EMG signal variability and the electrode replacement rather than the factorization algorithm. A 4-synergy model can be used for all examined tasks if only the global reconstruction has to be considered, while attention has to be paid in the choice of muscles if their specific reconstruction has to be performed. Moreover, we recommend focusing on the muscle synergy vectors or the temporal activity patterns rather than the number of the synergies, when seeking at the identification of a neurophysiological index.

## Figures and Tables

**Table 1:** Range of number of synergies for each subject across the sessions related to each task.

Range of NoS					
Sbj	W	S	R	A	D
#1	4–6	4–5	4–6	4–5	3–6
#2	3–6	4–6	3–5	4–6	3–6
#3	3–5	3–6	3–4	3–4	3–6
#4	3–6	3–6	3–4	3–5	3–6
#5	4–5	3–6	4–5	3–6	4–6
#6	3–5	3–6	3–4	3–5	3–5
#7	3–6	4–6	3–5	3–5	3–6
#8	3–4	3–6	3–5	4–6	3–6

**Table 2:** Interclass correlation coefficient (ICC) evaluated among sessions of variability account for values in the 4-synergy model for all tasks and for global EMG matrix and each muscle. Cells indicate the range of ICC values.

Interclass correlation coefficient (ICC)													
Task	Muscles												
	Global	TFL	REF	VLAT	VMED	PERO	TA	SOL	GLU	SEMT	BIF	LGAS	MGAS
W	**0.79**	**0.76**	**0.84**	**0.83**	**0.81**	***0.53***	**0.90**	**0.89**	**0.81**	***0.54***	***0.56***	***0.60***	**0.92**
S	**0.93**	**0.90**	**0.94**	**0.91**	**0.87**	*0.68*	**0.80**	**0.80**	**0.89**	**0.85**	**0.97**	*0.74*	**0.80**
R	**0.94**	**0.87**	*0.70*	**0.93**	**0.80**	**0.97**	**0.95**	**0.88**	**0.78**	**0.88**	*0.70*	**0.95**	**0.93**
A	**0.94**	**0.83**	**0.77**	*0.61*	**0.78**	**0.75**	**0.85**	*0.73*	**0.85**	**0.96**	**0.92**	**0.82**	**0.88**
D	**0.93**	**0.89**	**0.91**	**0.86**	*0.66*	**0.97**	**0.96**	**0.90**	**0.92**	*0.64*	**0.87**	**0.89**	**0.78**

Poor; ***fair***; *good*; **excellent**.

**Table 3:** Maximum and minimum and related standard deviation (SD) of the ^WW^VAF_glo_ and ^WW^VAF_loc_ related to each task.

Task		^WW^VAF_glo_ (%)	^WW^VAF_loc_ (%)											
			TFL	REF	VLAT	VMED	PERO	TA	SOL	GLU	SEMT	BIF	LGAS	MGAS
W	Min	92.1 (0.8)	79.7 (5.0)	81.8 (8.8)	83.7 (9.3)	77.9 (6.9)	79.5 (3.7)	71.8 (1.6)	87.5 (3.2)	79.2 (8.1)	86.6 (7.7)	78.6 (5.5)	91.3 (3.1)	86.5 (4.1)
	Max	96.3 (0.3)	96.7 (3.0)	98.2 (0.6)	99.3 (0.3)	98.6 (0.7)	98.5 (0.8)	98.4 (0.8)	98.9 (0.3)	97.4 (1.3)	99.3 (0.2)	99.2 (0.5)	99.3 (0.2)	98.9 (0.5)
S	Min	90.4 (1.0)	79.5 (8.4)	76.4 (9.5)	78.3 (7.0)	77.6 (5.4)	78.4 (8.2)	71.6 (4.9)	90.1 (3.1)	74.8 (7.4)	83.8 (9.6)	72.7 (2.6)	83.0 (3.8)	88.1 (9.6)
	Max	95.8 (0.8)	97.3 (0.7)	98.4 (0.7)	98.5 (0.7)	98.9 (0.5)	97.5 (0.4)	95.6 (2.2)	97.6 (1.1)	97.2 (1.3)	97.8 (1.5)	98.5 (0.4)	99.0 (0.5)	97.7 (1.6)
R	Min	94.1 (0.7)	88.2 (7.0)	88.3 (5.3)	90.2 (3.1)	88.9 (9.3)	89.7 (3.1)	73.2 (2.7)	94.2 (1.8)	78.7 (7.0)	90.8 (2.5)	91.0 (4.8)	80.6 (5.2)	93.2 (2.4)
	Max	98.4 (0.5)	98.6 (0.7)	98.8 (0.4)	99.4 (0.2)	99.5 (0.2)	99.2 (0.2)	99.6 (0.3)	99.4 (0.3)	98.8 (0.5)	99.5 (0.1)	99.0 (0.4)	99.6 (0.2)	99.6 (0.3)
A	Min	91.8 (0.9)	73.4 (1.1)	87.0 (9.3)	96.7 (0.6)	96.2 (1.0)	92.9 (1.5)	90.4 (2.0)	91.1 (7.1)	75.8 (8.6)	77.8 (2.2)	77.0 (8.7)	89.6 (5.5)	95.0 (1.5)
	Max	97.1 (0.4)	97.8 (0.7)	98.9 (0.6)	98.9 (0.4)	99.0 (0.3)	99.0 (0.4)	98.5 (1.3)	98.6 (0.5)	95.8 (1.7)	99.6 (0.3)	99.5 (0.2)	99.2 (0.3)	98.8 (0.2)
D	Min	91.7 (1.2)	76.6 (6.0)	89.4 (5.9)	93.0 (2.2)	94.6 (3.6)	75.1 (3.1)	77.6 (7.9)	74.2 (0.3)	73.4 (3.4)	90.0 (3.8)	77.6 (9.7)	78.5 (6.2)	82.9 (9.7)
	Max	96.4 (0.4)	96.7 (1.3)	98.0 (0.7)	98.7 (0.4)	98.4 (1.0)	98.2 (1.2)	99.1 (0.3)	98.2 (0.7)	98.5 (0.7)	98.0 (0.9)	97.2 (0.5)	96.9 (1.3)	96.9 (1.7)

**Table 4:** Maximum and minimum and related standard deviation (SD) of ^WW^cos_sim_ related to each task. The overall means evaluated considering all the synergies together are reported in italic.

Task		Muscle synergies				
		I	II	III	IV	Overall
W	Min	0.84 (0.10)	0.84 (0.11)	0.84 (0.10)	0.82 (0.24)	*0.92 (0.08)*
	Max	0.98 (0.01)	0.99 (0.00)	0.99 (0.01)	0.99 (0.00)	
S	Min	0.83 (0.12)	0.80 (0.08)	0.82 (0.15)	0.77 (0.11)	*0.90 (0.09)*
	Max	0.98 (0.01)	0.98 (0.01)	0.98 (0.01)	0.98 (0.01)	
	Min	0.81 (0.13)	0.73 (0.14)	0.81 (0.17)	0.82 (0.10)	*0.92 (0.09)*
R						
	Max	0.98 (0.01)	0.99 (0.01)	0.97 (0.01)	0.99 (0.00)	
	Min	0.78 (0.16)	0.87 (0.07)	0.87 (0.10)	0.79 (0.15)	*0.94 (0.07)*
A						
	Max	0.99 (0.00)	0.99 (0.01)	0.99 (0.01)	0.99 (0.01)	
	Min	0.71 (0.13)	0.73 (0.09)	0.80 (0.10)	0.73 (0.12)	*0.89 (0.09)*
D						
	Max	0.98 (0.01)	0.98 (0.01)	0.97 (0.02)	0.98 (0.01)	

**Table 5:** Maximum and minimum and related standard deviation (SD) of ^WW^*R*^2^ related to each task. The overall means evaluated considering all the synergies together are reported in italic.

Task		Muscle synergies				
		I	II	III	IV	Overall
W	Min	0.90 (0.07)	0.88 (0.08)	0.80 (0.09)	0.80 (0.07)	*0.93 (0.06)*
	Max	0.99 (0.01)	0.98 (0.01)	0.96 (0.04)	0.95 (0.04)	
S	Min	0.91 (0.05)	0.82 (0.03)	0.88 (0.10)	0.87 (0.07)	*0.92 (0.03)*
	Max	0.98 (0.02)	0.99 (0.02)	0.98 (0.01)	0.96 (0.03)	
	Min	0.85 (0.05)	0.90 (0.03)	0.88 (0.07)	0.86 (0.05)	*0.94 (0.03)*
R						
	Max	0.99 (0.01)	0.99 (0.02)	0.98 (0.01)	0.98 (0.02)	
	Min	0.91 (0.06)	0.84 (0.03)	0.82 (0.04)	0.90 (0.05)	*0.92 (0.03)*
A						
	Max	0.98 (0.01)	0.99 (0.00)	0.99 (0.02)	0.99 (0.02)	
	Min	0.88 (0.05)	0.84 (0.02)	0.85 (0.02)	0.84 (0.04)	*0.91 (0.06)*
D						
	Max	0.99 (0.02)	0.99 (0.01)	0.96 (0.03)	0.96 (0.04)	

**Table 6:** Minimum and maximum and relative standard deviation (SD) of the ^WB^VAF_glo_ and ^WB^VAF_loc_ related to each task.

Task		^WB^VAF_glo_ (%)	^WB^VAF_loc_ (%)											
			TFL	REF	VLAT	VMED	PERO	TA	SOL	GLU	SEMT	BIF	LGAS	MGAS
W	Min	93.0 (1.4)	88.0 (6.6)	85.6 (5.2)	89.2 (3.7)	83.3 (7.2)	85.1 (4.8)	78.1 (6.0)	92.4 (5.1)	85.7 (9.8)	92.9 (6.3)	87.8 (7.3)	95.3 (1.2)	90.6 (4.0)
	Max	95.2 (1.6)	95.1 (3.8)	97.7 (0.8)	96.9 (3.4)	98.2 (1.0)	97.3 (1.9)	93.4 (2.9)	98.1 (1.0)	95.4 (2.5)	98.6 (1.0)	98.0 (1.3)	98.6 (0.9)	97.9 (1.8)
S	Min	91.9 (1.9)	90.8 (4.2)	82.3 (8.8)	86.1 (6.4)	85.2 (6.3)	80.7 (8.4)	78.1 (7.8)	91.0 (4.9)	83.5 (8.7)	89.0 (6.9)	74.2 (4.8)	88.8 (9.7)	90.1 (6.8)
	Max	95.5 (0.7)	96.9 (1.3)	97.6 (1.2)	97.6 (1.6)	98.9 (0.4)	96.2 (2.1)	93.0 (5.1)	96.9 (1.8)	95.5 (2.2)	96.9 (1.3)	97.3 (2.0)	96.3 (1.7)	96.9 (1.9)
R	Min	95.1 (1.1)	90.7 (3.9)	91.8 (4.9)	93.9 (3.7)	94.3 (3.7)	91.5 (3.9)	78.7 (6.1)	95.7 (2.1)	87.6 (8.3)	92.6 (4.1)	94.0 (3.8)	88.7 (9.0)	95.0 (3.1)
	Max	97.8 (0.7)	97.5 (1.4)	98.1 (0.6)	99.0 (0.8)	99.0 (0.5)	98.8 (0.7)	99.5 (0.6)	99.2 (0.4)	98.3 (1.1)	98.0 (1.7)	97.3 (1.3)	99.4 (0.3)	99.2 (1.1)
A	Min	93.5 (1.8)	80.7 (9.6)	92.7 (7.3)	97.8 (1.2)	97.3 (1.2)	95.0 (1.9)	92.8 (5.0)	94.9 (4.9)	81.6 (7.5)	81.3 (7.6)	84.0 (8.5)	92.4 (3.0)	96.6 (1.6)
	Max	96.4 (0.5)	95.6 (2.2)	98.1 (0.8)	98.8 (0.7)	98.6 (0.7)	97.9 (1.6)	97.9 (2.0)	98.1 (0.8)	92.9 (6.2)	99.0 (0.8)	99.2 (0.5)	98.8 (0.6)	98.7 (0.5)
D	Min	92.5 (1.0)	81.3 (6.2)	89.7 (6.0)	95.1 (2.4)	96.2 (2.4)	77.8 (5.6)	81.3 (8.6)	82.1 (9.0)	80.3 (7.4)	92.7 (4.3)	83.4 (9.3)	82.6 (7.9)	88.2 (8.6)
	Max	96.1 (0.6)	95.6 (2.2)	97.2 (0.9)	98.0 (0.9)	97.6 (1.0)	96.9 (1.8)	98.4 (0.7)	96.1 (2.0)	96.8 (2.5)	96.4 (2.0)	96.4 (2.6)	96.5 (1.2)	96.1 (2.1)

**Table 7:** Maximum and minimum and relative standard deviation (SD) of the ^WB^cos_sim_ related to each task. The overall means evaluated considering all the synergies together are reported in italic.

Task		Muscle synergies				
		I	II	III	IV	Overall
W	Min	0.90 (0.00)	0.87 (0.05)	0.87 (0.06)	0.84 (0.01)	*0.92 (0.04)*
	Max	0.96 (0.02)	0.97 (0.01)	0.99 (0.01)	0.96 (0.02)	
S	Min	0.89 (0.03)	0.88 (0.03)	0.91 (0.01)	0.86 (0.04)	*0.94 (0.04)*
	Max	0.98 (0.00)	0.98 (0.01)	0.98 (0.01)	0.97 (0.02)	
	Min	0.90 (0.08)	0.91 (0.04)	0.89 (0.03)	0.92 (0.05)	*0.95 (0.04)*
R						
	Max	0.98 (0.01)	0.99 (0.01)	0.98 (0.00)	0.99 (0.00)	
	Min	0.93 (0.05)	0.90 (0.06)	0.91 (0.03)	0.95 (0.01)	*0.95 (0.03)*
A						
	Max	0.98 (0.02)	0.98 (0.01)	0.98 (0.01)	0.99 (0.01)	
	Min	0.91 (0.06)	0.91 (0.02)	0.86 (0.04)	0.88 (0.02)	*0.94 (0.04)*
D						
	Max	0.98 (0.01)	0.98 (0.01)	0.96 (0.02)	0.97 (0.02)	

**Table 8:** Maximum and minimum and relative standard deviation (SD) of the ^WB^*R*^2^ related to each task. The overall means evaluated considering all the synergies together are reported in italic.

Task		Muscle synergies				
		I	II	III	IV	Overall
W	Min	0.91 (0.05)	0.90 (0.05)	0.86 (0.08)	0.86 (0.04)	*0.90 (0.04)*
	Max	0.95 (0.04)	0.92 (0.06)	0.91 (0.07)	0.91 (0.05)	
S	Min	0.91 (0.05)	0.85 (0.03)	0.90 (0.02)	0.87 (0.07)	*0.90 (0.04)*
	Max	0.94 (0.03)	0.90 (0.01)	0.93 (0.04)	0.90 (0.05)	
	Min	0.86 (0.04)	0.90 (0.02)	0.90 (0.07)	0.88 (0.03)	*0.91 (0.04)*
R						
	Max	0.93 (0.01)	0.98 (0.01)	0.94 (0.02)	0.93 (0.05)	
	Min	0.90 (0.03)	0.86 (0.05)	0.86 (0.02)	0.89 (0.04)	*0.90 (0.05)*
A						
	Max	0.95 (0.03)	0.96 (0.03)	0.91 (0.02)	0.99 (0.01)	
	Min	0.92 (0.01)	0.87 (0.05)	0.87 (0.05)	0.85 (0.05)	*0.90 (0.05)*
D						
	Max	0.98 (0.00)	0.91 (0.01)	0.94 (0.05)	0.91 (0.05)	

**Table 9:** Mean value and standard deviation (SD) of ^B^VAF_glo_ and ^B^VAF_loc_ related to each task. Differences among muscles are reported in superscript.

Task	^B^VAF_glo_ (%)	^B^VAF_loc_ (%)												
		TFL (1)	REF (2)	VLAT (3)	VMED (4)	PERO (5)	TA (6)	SOL (7)	GLU (8)	SEMT (9)	BIF (10)	LGAS (11)	MGAS (12)	
W	94.1 (1.4)	91.8 (2.8)	90.4 (3.7)	94.0 (2.4)	90.2 (4.2)	92.2 (4.7)	86.5 (4.8)	96.3 (1.8)	90.8^11^ (3.2)	95.1 (2.3)	93.9 (3.9)	97.1^8^ (1.2)	95.3 (2.1)
S	93.9 (1.7)	92.5 (4.0)	92.9 (5.1)	92.3 (4.4)	91.0 (4.4)	91.0 (4.8)	84.3^12^ (4.6)	94.1 (2.0)	91.3 (3.8)	94.3 (2.4)	90.4 (7.6)	93.9 (2.5)	94.3^6^ (2.0)
R	96.6 (1.3)	93.8 (2.6)	95.9 (2.5)	97.8 (1.6)	97.2 (1.7)	97.2 (2.5)	90.8 (7.0)	98.1 (1.2)	91.9 (3.5)	96.1 (1.8)	96.1 (1.1)	97.6 (3.6)	98.0 (1.4)
A	95.4 (1.4)	86.7^2,3,4,5,6,7,12^ (4.3)	96.0^1,8^ (1.6)	98.1^1,8^ (0.3)	97.9^1,8^ (0.6)	96.8^1,8^ (0.9)	95.0^1^ (1.9)	97.1^1,8^ (1.0)	88.8^2,3,4,5,7,12^ (3.6)	91.2 (6.9)	91.8 (4.9)	95.1 (2.1)	97.7^1,8^ (0.7)
D	94.5 (1.6)	89.1 (5.3)	95.2 (2.4)	96.8 (0.9)	96.9 (0.6)	91.4 (6.2)	92.9 (6.0)	91.1 (4.8)	88.5 (5.1)	94.7 (1.4)	92.0 (3.9)	89.4 (4.4)	92.7 (2.5)

**Table 10:** Mean value and standard deviations (SDs) related to the ^B^COS_sim_ evaluated synergy by synergy for each task. Differences between pair of synergies in each task are reported in superscript with the relative roman number; ∗ stands for statistical differences among all synergies. The overall means evaluated considering all the synergies together are reported in italic.

Task	Muscle synergies				
	I	II	III	IV	Overall
W	0.84^III^ (0.06)	0.83^III^ (0.07)	0.90^∗^ (0.05)	0.83^III^ (0.06)	*0.85 (0.07)*
S	0.83 (0.07)	0.84 (0.09)	0.82 (0.09)	0.80 (0.11)	*0.82 (0.08)*
R	0.84^IV^ (0.09)	0.83^IV^ (0.10)	0.83^IV^ (0.07)	0.90^∗^ (0.06)	*0.85 (0.08)*
A	0.82^II^ (0.10)	0.90^I^ (0.05)	0.85 (0.07)	0.86 (0.08)	*0.86 (0.08)*
D	0.79^III^ (0.08)	0.81^III^ (0.09)	0.90^∗^ (0.05)	0.81^III^ (0.05)	*0.83 (0.08)*

**Table 11:** Mean value and standard deviations (SDs) related to the ^B^*R*^2^ evaluated synergy by synergy for each task. Differences between pair of synergies in each task are reported in superscript with the relative roman number; ^∗^ stands for statistical differences among all synergies. The overall means evaluated considering all the synergies together are reported in italic.

Task	Muscle synergies				
	I	II	III	IV	Overall
W	0.89^III,IV^ (0.05)	0.90^III,IV^ (0.03)	0.84^I,II^ (0.06)	0.86^I,II^ (0.07)	*0.87 (0.06)*
S	0.85^II^ (0.08)	0.91^∗^ (0.04)	0.85^II^ (0.06)	0.87^II^ (0.05)	*0.87 (0.06)*
R	0.84^II^ (0.04)	0.92^∗^ (0.07)	0.87^II^ (0.04)	0.86^II^ (0.06)	*0.87 (0.06)*
A	0.89^II^ (0.04)	0.84^I,IV^ (0.06)	0.86 (0.08)	0.89^II^ (0.05)	*0.87 (0.06)*
D	0.90^∗^ (0.04)	0.84^I,IV^ (0.06)	0.84^I,IV^ (0.05)	0.79^∗^ (0.06)	*0.84 (0.06)*
